# Screening for Effects of Inhaled Nanoparticles in Cell Culture Models for Prolonged Exposure

**DOI:** 10.3390/nano11030606

**Published:** 2021-02-28

**Authors:** Claudia Meindl, Kristin Öhlinger, Verena Zrim, Thomas Steinkogler, Eleonore Fröhlich

**Affiliations:** 1 Center for Medical Research, Medical University of Graz, Stiftingtalstr. 24, 8010 Graz, Austria; Claudia.meindl@medunigraz.at (C.M.); Kristin.oehlinger@medunigraz.at (K.Ö.); verena.zrim@medunigraz.at (V.Z.); 2 Institute of Chemical Technologies and Analytics, Vienna University of Technology, Getreidemarkt 9/164, 1060 Vienna, Austria; thomas.steinkogler@tuwien.ac.at

**Keywords:** nanotoxicity, respiratory exposure, in vitro models, polystyrene particles, macrophages, cytotoxicity, repeated exposure, air–liquid interface, proinflammatory effects

## Abstract

Respiratory exposure of humans to environmental and therapeutic nanoparticles repeatedly occurs at relatively low concentrations. To identify adverse effects of particle accumulation under realistic conditions, monocultures of Calu-3 and A549 cells and co-cultures of A549 and THP-1 macrophages in the air–liquid interphase culture were exposed repeatedly to 2 µg/cm^2^ 20 nm and 200 nm polystyrene particles with different functionalization. Particle accumulation, transepithelial electrical resistance, dextran (3–70 kDa) uptake and proinflammatory cytokine secretion were determined over 28 days. Calu-3 cells showed constant particle uptake without any change in barrier function and cytokine release. A549 cells preferentially ingested amino- and not-functionalized particles combined with decreased endocytosis. Cytokine release was transiently increased upon exposure to all particles. Carboxyl-functionalized demonstrated higher uptake and higher cytokine release than the other particles in the A549/THP-1 co-cultures. The evaluated respiratory cells and co-cultures ingested different amounts and types of particles and caused small (partly transient) effects. The data suggest that the healthy cells can adapt to low doses of non-cytotoxic particles.

## 1. Introduction

The respiratory pathway, more specifically the deep lung, represents the most permeable and sensitive epithelial barrier of the human body. Despite protection by mechanisms like the beating of cilia of bronchial epithelial cells (mucociliary clearance) and phagocytosis by alveolar macrophages, particles reach intendedly (for drug treatment) or unintendedly (airborne particulate matter) the alveolar region of the deep lung. The contact, in general, is repeated and at low doses. If the deposited material cannot be degraded, accumulation may occur. Nonbiodegradable nanoparticles include iron, lead, zinc, copper, cobalt, nickel, chromium, manganese, and tin nanoparticles in brakes, tires, road dust, in the chemical industry and metalworking and carbon nanotubes, silica nanoparticles, polymeric nanoparticles, and polymersomes for drug delivery [1,2]. For these nanoparticles, conventional toxicity testing using much higher than realistic doses for 24–72 h of exposure is not the most appropriate testing model, and cellular models that enable longer exposure times are needed.

Due to the good reproducibility of the data and the easy handling, cell lines cultured in plastic dishes are routinely used models for cytotoxicity screening. Disadvantages compared to primary cells are the lower differentiation state and higher proliferation rate of the cells, which limit the physiological relevance of the data. By changing the culture conditions, from plastic to other surfaces, by culture in 3D, by addition of mechanical forces, or by the culture at the air–liquid interface, cell differentiation can be increased. The increased differentiation state often results in decreased proliferation, which enables the longer study of the same population of cells [3]. Calu-3 cells are routinely used for permeability studies of inhaled compounds. They are derived from lung adenocarcinoma, resemble bronchial epithelial cells and form monolayers when grown on membrane inserts [4]. In these cultures, constant transepithelial electrical resistance (TEER) values were observed for 14–70 days [5]. By culture on membrane inserts at the air–liquid interface (ALI), Calu-3 cells produce mucus and could be used to assess nanoparticle effects over 28 days [6]. To assess toxicity in the deep lung (alveoli), A549 cells are the most commonly used model. Depending on the subtype and the culture condition, they can form monolayers or multiple layers on Transwell membranes [7]. The more slowly proliferating subtype (doubling time of 24 h as used in this study) cultured in ALI with a minimum amount of medium in the basolateral compartment of the Transwell to reduce hydrostatic pressure induces the formation of monolayers [8]. For realistic in vitro testing, it would be good to include also other relevant cell types of the lungs. The culture of alveolar epithelial cells together with macrophages, endothelial cells, dendritic cells, mast cells, and combinations thereof have been established (for more information, for instance, [9]). These models are stable regarding their cellular composition only for few days due to differences in medium requirements, proliferation rates of the cells, and the variable ability to tolerate ALI condition. Variation of the cell composition in co-cultures has, for instance, been studied for co-cultures of Caco-2/HT-29 cells as a model for the intestinal lining [10]. The initial seeding ratio of 3:7 had changed after 14 days to 8:2 due to the higher proliferation rate of the HT-29 cells. By variation of the cell seeding ratio and the cell culture media, stable co-cultures may be obtained. The ratio of alveolar cells to macrophages in the human lung is 9:1 [11], and a similar ratio would be needed for representative testing.

It is not expected that the low exposure doses, which have been determined for humans, acutely damage cells. However, particles may accumulate upon repeated exposure and alter physiologically relevant cell functions. Endocytosis and transport are important functions of endothelial and alveolar epithelial cells. Dextran polymers of different molecular weights (3 and 70 kDa) are one of the most commonly used endocytotic markers [12]. 70 kDa dextran is a good marker for macropinocytosis, whereas 10 kDa dextran and smaller molecules are better for probing general fluid-phase endocytosis (macropinocytic and micropinocytic processes). A549 cells have low macropinocytosis rates, which can be markedly upregulated, for instance, upon damage, e.g., exposure to bacteria [13]. Further, the absence of inflammation is crucial for the normal function of the lung. Nanoparticles may act proinflammatory, and cytokines, e.g., interleukin (IL) 6 and IL-8, were shown to be sensitive markers for nanoparticle toxicity [14].

Several studies measured the exposure levels of humans at the workplace. Based on some data, a deposited mass of 2.8 ng/cm^2^ per day or a lifetime exposure of 12.4–46.5 µg/cm^2^ was assumed [15,16]. Lung exposure levels to cellulose nanocrystals and carbon nanotubes were indicated with 0.14–1.57 µg/cm^2^ and 0.24–2.4 µg/cm^2^, respectively [17,18]. For the treatment of pulmonary cancer, 0.5–0.86 µg/cm^2^ lung surface is needed [19]. To deliver the desired amount of particles to cultured cells, various methods have been used [20]. In addition to commercial systems available from Cultex® Technology GmbH and VITROCELL Systems GmbH and manual devices for aerosolization (MicroSprayer® and Dry Powder Insufflator™), which are provided by Bio Jane Trading Limited, many researchers developed their own exposure devices. Delivery as an aerosol with all devices may result in variable aerosolization, adherence to walls, and difficulties in the determination of the delivered dose. One option to avoid these problems, which was used in this study, is particle suspension in cell culture medium or simulated lung fluid (SLF). SLF, which mimics the composition of the alveolar surfactant, consists of phosphate-buffered saline (PBS) with 0.02% of the lipid dipalmitoylphosphatidylcholine (DPPC) and is a medium for dissolution testing of inhaled drugs [21]. To maintain air–liquid interface conditions, the application should be performed in the smallest volume that covers all cell surfaces. Commercially available fluorescently labeled polystyrene particles were used as model particles to study the effects of accumulation. Although plastic particles, in general, are biocompatible and do not contain heavy metal ions or polyaromatic hydrocarbons like other nonbiodegradable particles, these particles caused adverse effects in cells and animal models [22]. These effects may be caused by accumulation, which can be monitored based on fluorescence. Polystyrene particles are very suitable because by the inclusion of fluorescent dye inside the particle, the fluorochrome is protected, and the labeling does not affect the surface functionalization of the particles.

This study aims to answer the question if repeated exposure to low doses of nonbiodegradable and biocompatible nanoparticles causes subtle damage in specific parts of the respiratory system. As particles in the upper respiratory tract get into direct contact only with bronchial epithelial cells, a monoculture of Calu-3 bronchial epithelial cells in ALI culture was used as representative model. In the deep lung, particles may be ingested by alveolar epithelial cells and by alveolar macrophages, and, therefore, co-cultures of A549 alveolar epithelial cells and THP-1 macrophages were established to evaluate the effects. To find out if macrophages modulate particle effects on A549 cells, particle action was also studied in A549 monocultures. Cultured cells were exposed to low levels of polystyrene particles with different surface functionalization applied in a small volume of SLF. These particles were carboxyl-functionalized polystyrene particles in sizes of 20 nm and 200 nm (CPS20 and CPS200), 200 nm non-functionalized (plain) polystyrene particles (PPS200), and 200 nm amine-functionalized polystyrene particles (AMI200). Barrier function in Calu-3 cells, endocytosis in A549 cells, and particle uptake, secretion of IL 6 and IL 8 in all cultures were determined after repeated particle exposure for 28 days.

## 2. Materials and Methods

### 2.1. Cells

A549 cells obtained from Deutsche Sammlung für Mikroorganismen und Zellkulturen GmbH (Braunschweig, Germany) were cultured in Dulbecco’s modified Eagle’s medium (DMEM), 2 mM L-glutamine, 1% penicillin/streptomycin (P/S) and 10% fetal bovine serum (FBS) and passaged at regular intervals. Calu-3 cells were obtained from LGC Standards GmbH (Wesel, Germany) and cultured in MEM, 2 mM L-glutamine, 1% penicillin/streptomycin and 10% fetal bovine serum (FBS), and 1 mM sodium pyruvate. THP-1 was purchased from Cell Line Services (Eppelheim, Germany) and cultured in RPMI, 2 mM L-glutamine, 1% penicillin/streptomycin and 10% FBS.

### 2.2. Preparation of Calu-3 Cells

**Figure 1:**
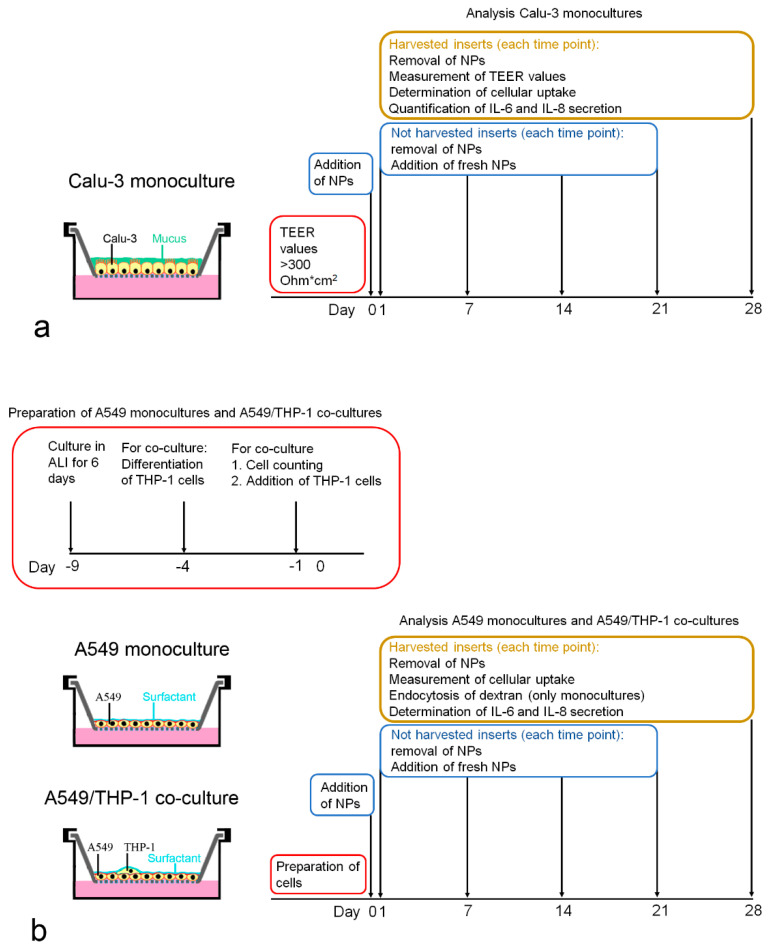
Schematic representation of Calu-3 monoculture (**a**), A549 monoculture and A549/THP-1 co-culture (**b**), preparation of the cells, particle exposures and analyses at each time point.

0.5 × 10^6^ cells were seeded in 500 µL MEM, 2% L-glutamine, 1% PS + 10% FBS on 12-well polyethylene terephthalate Transwell inserts (pore size 0.4 µm, Greiner Bio-one, Kremsmünster, Austria) with 1500 µL of the same medium in the basolateral compartment. Medium in the apical compartment was removed after 24 h, and the medium amount in the basolateral compartment was reduced to 500 µL, which was changed every 2 or 3 days. Cells were used for the experiments when they had reached a transepithelial electrical resistance (TEER) value of >300 Ω*cm^2^. Cell numbers were obtained after removal of the cells from the membrane by incubation with 100 µL trypsin-EDTA (0.05%) for 15 min at 37 °C, addition of 1400 µL MEM + 10% FBS, and followed by gentle scraping with the tip of a pipette. Cell number was determined using a Casy TT cell counter (OLS OMNI Life Science, Bremen, Germany). Particle exposures and analyses performed at each time point are summarized in Figure 1a.

### 2.3. Differentiation to THP-1 Macrophages

1.0 × 10^6^ THP-1 cells/mL were seeded in RPMI 1640 containing 10% FBS, 1% L-glutamine, and 1% P/S in flasks. For differentiation, 10 nM phorbol 12-myristate 13-acetate (PMA, Sigma-Aldrich, Vienna, Austria) was added to the media for 72 h, followed by changing the media to medium without PMA for another 24 h before use in the co-cultures. Cells were harvested by treatment with trypsin/EDTA (0.05%).

### 2.4. Monolayer Formation

Cell-grown inserts were fixed in 4% paraformaldehyde and embedded in paraffin using Tissue-Tek®VIP™ (SanovaPharma GesmbH, Gallspach, Austria). Radial sections of 2 µm were cut at a rotary microtome, stained with hemalaun, and viewed at an Olympus BX51 microscope.

### 2.5. Determination of Cell Number in A549 Monoculture

2.5 × 10^5^ A549 cells were seeded in 500 µL DMEM, 2% L-glutamine, 1% PS + 10% FBS on 12-well polyethylene terephthalate Transwell inserts (pore size 0.4 µm, Greiner Bio-one, Kremsmünster, Austria) with 1500 µL of the same medium in the basolateral compartment. Medium in the apical compartment was removed after 24 h, and the medium amount in the basolateral compartment was reduced to 500 µL. This time point was defined as d0. To monitor cell number over time, cell number was assessed in the following way on days 7, 14, 21, and 28. Cells were removed from the membrane by incubation with 100 µL trypsin-EDTA (0.05%) for 15 min at 37 °C, addition of 1400 µL DMEM + 10% FBS, and followed by gentle scraping with the tip of a pipette. Cell number was determined by Casy TT cell counter (OLS OMNI Life Science). At time points later than 7 days, these measurements, in combination with flow cytometry data and morphological analysis, indicated that a fraction of the cells was dead. Therefore, cell counting was omitted at later time points.

### 2.6. A549 Monoculture and A549/THP-1 Co-Culture for Particle Exposure Studies

Pilot experiments showed that using a lower number of A549 cells in combination with precultivation prior to the addition of THP-1 cells reduced the risk of monolayer detachment at later time points. Therefore, the protocol given in the previous section on the determination of cell numbers was slightly modified: a lower number of cells/inserts were seeded (0.8 × 10^5^), and cells were cultured for 9 days in ALI prior to the addition of the THP-1 cells. Instead of DMEM + 10% FBS, the basal compartment was filled with RPMI 1640 + 10% FBS because cell proliferation was lower with this medium. To add THP-1 cells in a definite ratio to the A549 cells, the average number of A549 cells was determined in duplicates and the number of added THP-1 macrophages based on the counted A549 cells. Preparation of the monocultures and A549/THP-1 co-cultures for the particle exposures and analyses performed at each time point are summarized in Figure 1b.

### 2.7. Identification of Macrophages

THP-1 macrophages were identified by anti-CD45 staining according to the following protocol. Cells were fixed for 15 min in 4% paraformaldehyde, followed by permeabilization for 20 min with 0.1% Triton X in PBS. Incubation with (1) anti-human CK18 antibody (rabbit, 1:1000, Sigma-Aldrich) in antibody diluent (DAKO, Vienna, Austria) for 1.5 h at RT, (2) anti-rabbit Alexa488-labeled antibody (rabbit, 1:500, Thermo Fisher Scientific, Vienna, Austria) + 1 µg/mL Hoechst 33342 for 30 min at RT and (3) anti-human CD45 APC-Cy7 (mouse, 1:500, BD Biosciences) at 1 h at RT in antibody diluent. Between all steps, inserts were rinsed three times with PBS. For negative controls, the primary antibodies were substituted by mouse IgG (1:1000, Linaris, Dossenheim, Germany).

To provide a ratio of A549:THP-1 cells of ~10:1 over prolonged cultivation time, THP-1 cells were added in A549:THP-1 ratios of 1:0.4, 1:1, 1:1.5, and 1:2 to the A549 cells and cultures stained with CD45 antibody and Hoechst 33342. Images (16 regions of interest representing 6,250,000 µm^2^) were taken at a motorized Nikon Ti2 E inverted microscope with a C2 confocal system (Nikon CEE GmbH, Vienna, Austria) using an Andor Zyla VSC-08691 camera and the ratio of the number of CD45-immunoreactive cells to the number of nuclei identified by Hoechst 33342 staining determined (Table 1).

**Figure 2:**
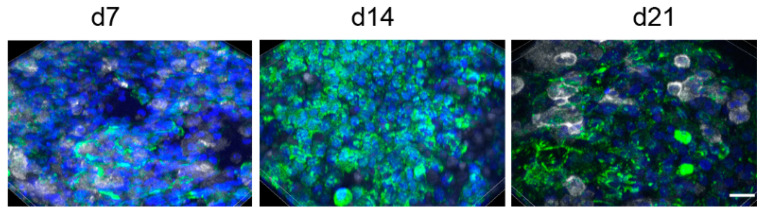
A549 were identified by anti-CK18 antibody (green) and THP-1 cells by anti-CD45 staining (white). Nuclei are counterstained with Hoechst 33342 (blue). Z-stack images of the A549/THP-1 co-cultures were reconstructed and shown as 3D projections. Scale bar: 20 µm.

A seeding ratio of A549:THP-1 of 1:1 was chosen for the experiments resulted in the following ratios of A549 to THP-1 cells at 7 (9 ± 3:1), 14 (8 ± 3:1), and 21 days (10 ± 7:1). Evaluation of 28 days was complicated because staining of cells with CD45-antibody became less distinct over the culture time (Figure 2). The figure also illustrates that, despite performing the same protocol, staining intensities varied between the time points.

### 2.8. Particles

Red carboxyl-functionalized particles 20 nm and 200 nm (FluoSpheres® carboxylate modified microspheres, Invitrogen), 200 nm red amine-functionalized particles (FluoSpheres® amine-modified microspheres, Invitrogen) and non-functionalized 200 nm red-dyed polystyrene particles (Fluoro-Max R200, Thermo Fisher Scientific) were used. The particles were characterized in DMEM w/o FBS and in SLF. SLF was prepared by adding 385 μL stock solution of DPPC (13 mg/mL in ethanol) to 25 mL of a preheated (37 °C) DPBS with calcium and magnesium (Thermo Fisher Scientific).

### 2.9. Preparation of Particle Suspensions

To determine the minimal amount of fluid that covers all cells of the Transwell insert, cells were seeded in the same way as for the particle exposures and 10–50 µL PBS containing 0.5% trypan blue was applied. The area colored in blue was captured by Olympus digital camera E-PM1 attached to a stereomicroscope (SZX12, Olympus, Vienna, Austria). A volume of 40 µL was needed to create a homogenous blue cell surface. Dispersions of the particles were prepared from the commercial solutions diluted with DMEM (w/o FBS) or SLF. The dispersions were put into an Elmasonic S40 water bath (ultrasonic frequency, 37 kHz, 40 W, Elma) for 20 min prior to cell exposure.

### 2.10. Characterization of Particles

Particles were characterized in DMEM w/o FBS and PBS + 0.02% DPPC (SLF). The particles were suspended in the media and ultrasonicated for 10 min. Particles for characterization in DMEM w/o FBS were measured via photon correlation spectroscopy (PCS, Malvern Zetasizer, Malvern Instruments, Malvern, UK) equipped with a 532 nm laser. The zeta potential was measured by laser Doppler velocimetry (scattering angle of 17°) coupled with photon correlation spectroscopy (Zetasizer Nano ZS, Malvern Instruments) and calculated out of the electrophoretic mobility by applying the Henry equation. Characterization in PBS + 0.02% DPPC was determined at 50 µg/mL by dynamic light scattering (DLS) and electrophoretic light scattering (ELS) at a wavelength of 658 nm from a 40 mW single-frequency laser diode using LiteSizer 500 (Anton Paar, Graz, Austria). Three suspensions were prepared for every particle type, and each of them was measured three times with DLS (three data points per measurement) and three times with ELS (500 data points per measurement), respectively. It has been shown that, despite potential interference, DLS is an appropriate technique for the determination of the hydrodynamic size of fluorescent particles [23].

### 2.11. Stability of The Fluorescent Polystyrene Particles

The fluorescence of the labeled polystyrene particle was determined after different times of incubation because a stable signal is required to estimate accumulation. Particles solutions as used for the cell incubations were kept in the cell incubator and measured against the respective freshly prepared suspensions of CPS20, CPS200, AMI200, and PPS200 particles and exposure medium after 7 days, 14 days, 21 days and 28 days of incubation. The freshly prepared solutions were set as 100%. The 20 nm amine-functionalized particles (green dyed Estapor F2-XC, Merck Millipore, Paris, France), and the 20 nm plain polystyrene particles (Fluoro-Max G25, Thermo Scientific, Vienna, Austria), which originally were also planned for comparison, did not show stable fluorescence. The signal of the amine-modified particles steeply declined within one week, and that of the 20 nm non-functionalized plain particles showed large variations between the time points (data not shown). These particles were excluded from further analysis.

### 2.12. Semiquantification of Particle Uptake

Cells were exposed to 2 µg particles/insert contained in 40 µL SLF or DMEM w/o FBS. After the indicated time points, two inserts/conditions were rinsed three times with PBS and harvested, while fresh particle solutions were added after the rinsing to the remaining inserts. Cells were harvested using a combination of 0.05% trypsin/EDTA and gentle scraping with the tip of a pipette to remove all remaining cells from the membrane.

Fluorescence was read at Ex/Em wavelength of 584/612 nm (CPS20, CPS200, AMI200) and 544/612 nm (PPS200) in a black plate using a fluorescence plate reader (FLUOstar Optima, BMG Labortechnik, Ortenberg, Germany). A standard curve was prepared using the solutions that had been added as stock solution and diluting them with cell suspensions to account for interference of the cells with the fluorescent signal. The stock solutions used for the standard curve were kept at 37 °C for the incubation time of 24 h. Cell numbers and viability were determined using a CASY TT cell counter and analyzer system (OLS OMNI Life Science).

Uptake was calculated as cumulative uptake (% uptake) without any correction (based on fluorescence of the freshly prepared standard curve) and as% uptake divided by the number of applied doses.

To find out if particles crossed the epithelial barrier, fluorescence in the basolateral compartment was also determined. Dilutions of the particles in the medium were performed in the same way as for cellular uptake to use the same setting of the fluorescence plate reader. The only difference was that no cell suspension was added for the preparation of the standard curve.

### 2.13. Cytotoxicity Screening by Determination of Dehydrogenase (MTS Assay)

CellTiter 96® aqueous nonradioactive cell proliferation assay (Promega, Mannheim, Germany) was used according to the manufacturer’s instructions. In short, 100 µL of the combined MTS/PMS solution + 500 µL medium were added in each insert. Plates were incubated for up to 2 h at 37 °C in the cell incubator. Absorbance was read at 490 nm on a plate reader (SPECTRA MAX plus 384, ServoLab, Austria).

### 2.14. Measurement of Transepithelial Electrical Resistance (TEER) Values

TEER values were determined for all cultures of Calu-3 cells every 2–3 days with an EVOM STX-2-electrode (World Precision Instruments, Berlin, Germany). 0.5 mL MEM was added to the apical and 1.5 mL MEM to the basolateral compartment for TEER measurements. TEER values were calculated as follows:

TEER (Ω * cm^2^) = (Sample–blank resistance, given in Ω) * membrane area, given in cm^2^.

Blank resistance is defined as the resistance of the membrane without cells, and the membrane area for 12-well inserts is calculated to be 1.12 cm^2^/well. As TEER needs some time to recover, it was not measured for the 1 d exposures.

### 2.15. Endocytosis by Dextran Uptake

After the exposures, cells were rinsed in PBS and detached from the membrane using 100 µL trypsin/EDTA solution for 10 min at 37 °C. Subsequently, medium containing 0.25 mg/mL FITC-labeled dextran MW 3000, 0.5 mg/mL FITC-labeled dextran 10,000, 0.5 mg/mL FITC-labeled dextran 70,000 (Fisher Scientific, Vienna, Austria) was added and remaining cells on the insert membrane dislodged using the tip of a pipette. The cell suspension was kept for 60 min in the cell incubator to allow uptake of the dextran. After centrifugation for 5 min at 800 rpm, the supernatant was removed, cells resuspended in PBS and centrifugated again. This procedure was repeated three times. After the third centrifugation, cells were resuspended in PBS + 3% FBS, samples measured at an LSR-II (Becton Dickenson, Vienna, Austria) and analyzed by BD FACSDiva 8.0.1.

To differentiate between dextran binding and uptake, endocytosis was blocked by the addition of 25 mM NaN_3_ for Dextran 3000 and 50 mM for dextran 10,000 and 70,000 to the exposure medium and incubation on ice instead of 37 °C. Uptake of FITC-labeled dextran 3000 was reduced from 16.93 ± 5.93 to 0.15 ± 0.06, uptake of dextran 10,000 from 5.20 ± 1.08 to 0.20 ± 0.08, and that of dextran 70,000 from 2.33 ± 0.22 to 0.05 ± 0.06, suggesting that the contribution of unspecific binding to the plasma membrane to the signal was negligible.

These experiments were not performed with Calu-3 cells because no good signal was obtained in the untreated cells.

### 2.16. Endotoxin Detection

Since cytokine secretion may be induced by the presence of lipopolysaccharide (LPS), its absence must be shown in the samples. PYROGENT Ultra (sensitivity = 0.06 EU/mL, Szabo-Scandic, Vienna, Austria) was used for the endotoxin testing. The assay is very sensitive and indicated endotoxin in the freshly prepared medium. Therefore, all samples were tested in dilution when a negative result for the freshly prepared medium was obtained. The assay was performed according to the instructions given in the manual with samples tested in duplicates and the different endotoxin standards with *E*. *coli* strain 055:B5 in triplicates. For each time point, two inserts were tested for endotoxin. No experiments containing inserts with higher than basal (fresh medium) endotoxin content were included in the evaluation.

### 2.17. Cytokine Secretion

Medium from the basolateral compartments of all cultures was collected, and the release of IL-6 and IL-8 was determined using the human IL-6 ELISA set (BD OptEIA™) and the human IL-8 ELISA set (BD OptEIA™, BD Biosciences, Heidelberg, Germany) according to the protocol given by the producers. To induce IL-6 and IL-8 secretion, 24 h prior to the harvesting, 40 µL containing 20 µg LPS (positive control for A549 cells) or 100 µg LPS (positive control for Calu-3 cells), respectively, were added. Absorbance was read at 450 nm (with correction wavelength of 570 nm) on a SPECTRA MAX plus 384 photometers.

### 2.18. Statistics

Experiments were performed in triplicates and repeated at least two times. Data from all experiments were analyzed with one-way analysis of variance (ANOVA) followed by Tukey’s HSD post hoc test for multiple comparisons (SPSS 21 software). Results with *p*-values < 0.05 were considered to be statistically significant.

## 3. Results

### 3.1. Characterization of Polystyrene Particles (Hydrodynamic Size, Zeta Potential, Stability of the Fluorescence)

Description of the particles as indicated by the producer together with hydrodynamic sizes and zeta potentials suspended in the exposure fluids are summarized in Table 2. Size and zeta potentials of particles suspended in DMEM w/o have been reported previously [8,24]. Zeta sizes were in the negative range for CPS20, CPS200 and PPS200 particles and positive for AMI200 particles. Suspension in SLF increased particle size of CPS20 and AMI200 particles and shifted the zeta potential of CPS200 particles to less negative values.

**Figure 3:**
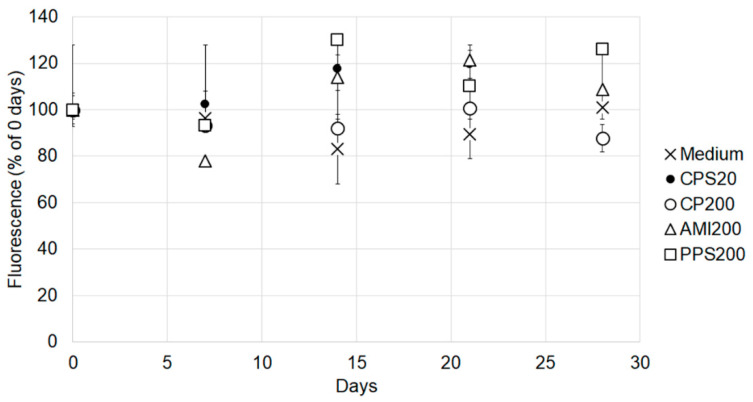
Fluorescence of particle suspensions stored in the cell incubator for up to 28 days compared to freshly prepared suspensions. The fluorescence signal of freshly prepared suspensions was defined as 100%.

To assess the stability of the fluorescence signal, particle suspensions at the same concentrations as used in the cell exposures were stored in the cell incubator for up to 28 days and fluorescence measured at the different time points in parallel to freshly prepared suspensions. Figure 3 shows that the particles maintained stable fluorescence over the entire incubation time.

### 3.2. Characterization of Long-Term Monocultures of Calu-3 Cultures, A549 Cultures, and A549/THP-1 Co-Cultures

As reported previously [6], there was little variation of the Calu-3 cell number (3.8–4.9 × 10^5^ cells/inserts over time). Radial sections of the cultures show little variation over time, except for the increased frequency of cell patches with multiple layers at d21 (Supplementary Materials, Figure S1). It may be assumed that at these cell patches, cells of the basal layer will not be exposed to particles. Basal average levels were 39–52 pg/mL for IL-6 and 24–52 pg/mL IL-8, and the LPS-induced values 482–605 pg/mL for IL-6 and 153–208 pg/mL for IL-8. TEER values as an indication for epithelial barrier function declined slightly from 513–612 Ω*cm^2^ at d0 to 418–469 Ω*cm^2^ at d28.

**Figure 4:**
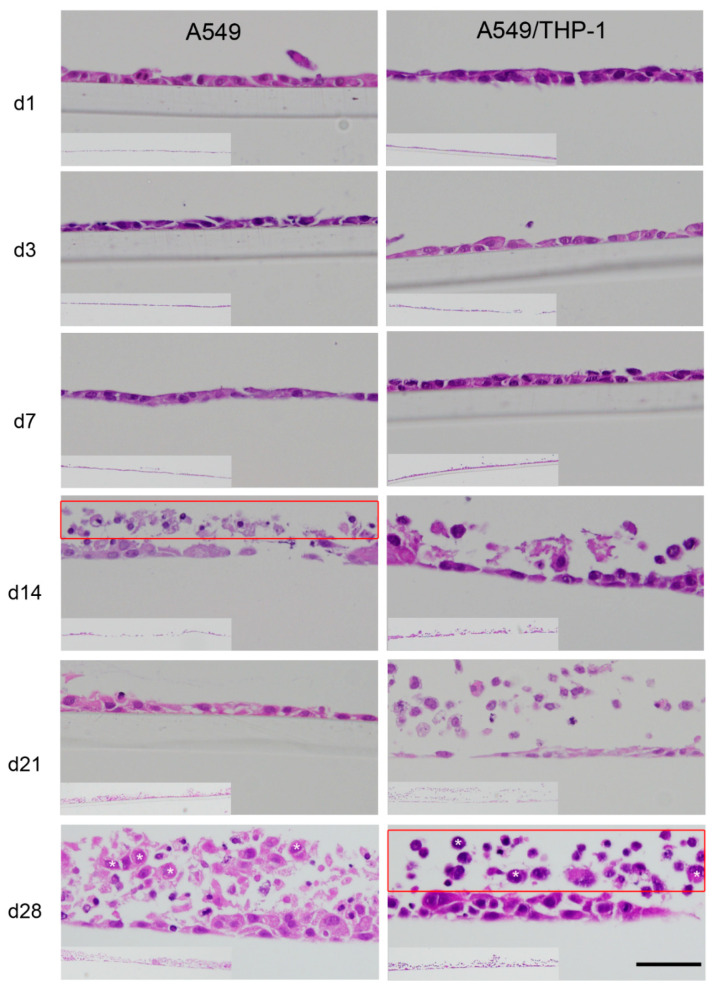
Radial section of A549 mono and A549/THP-1 co-cultures stained with hemalaun. After 14 days, dead cells arise on top of the monolayer. At 28 days, pronounced shedding of dead cells is seen. Inserts with lower magnification show regional variations. The scale bar indicates 50 µm in the higher and 500 µm in the lower magnifications (inserts).

Radial sections of the membrane-grown A549 cells show that starting at d14 necrotic cells are found on top of the A549 monolayer (Figure 4). At d28, more cells accumulate, and multilayers appear. According to the viability assay, the number of viable cells remained fairly constant with variation in the absorbance of the dehydrogenase activity (MTS) assay of 0.38–0.53 at d1–d28. This is also reflected by the interleukin levels, which were around 0–1.4 pg/mL for IL-6 and 6.7–25.5 pg/mL for IL-8 in the unstimulated cells. Upon stimulation, with LPS, IL-6 concentrations of 397–451 pg/mL and IL-8 concentrations of 207–273 ng/mL were detected. As also reported by other authors, the presence of DPPC in the incubation media did not change the basal secretion and the LPS-induced secretion after >6 h of cytokines by A549 and Calu-3 cells [25].

TEER values of A549 cells were not measured at all time points because they were already low (around 50 Ohm/cm^2^) at the start of the culture. The lack of tight barrier formation is in line with published data, e.g., [8].

Images from radial sections of the membrane-grown mono- and co-cultures (Figure 4) show necrotic cells starting at d14 and an increase of their number at the subsequent time points. The number of viable cells increased over the incubation time as the increase in absorbance values of the dehydrogenase activity (MTS) assay from 0.48–0.57 at d1–d14 to 0.72–0.74 at d21-d28 also showed. Basal cytokine secretion with 0.04–0.1 pg/mL of IL-6 and 6–20 ng/mL of IL-8 from d1–d21 was higher than in the A549 monocultures. The LPS-stimulated releases were 2–5 ng/mL for IL-6 and 348–497 ng/mL for IL-8 at d1–d21. At d28, however, cytokine secretion could no more be detected, neither in the unstimulated nor in the LPS-stimulated cultures.

### 3.3. Effect of Particles on Viability and Barrier Function

**Figure 5:**
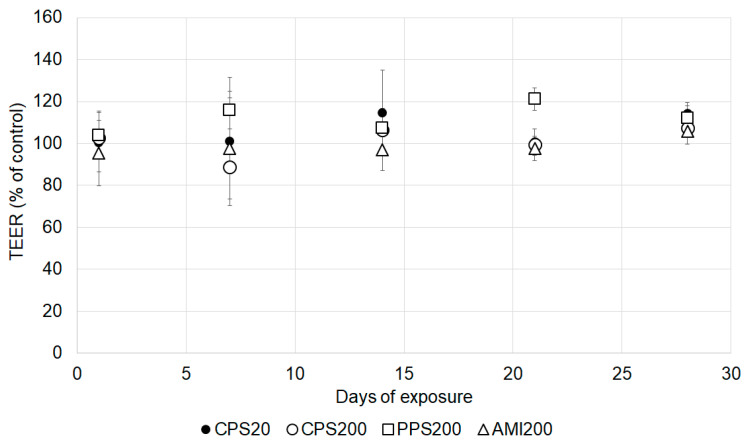
Relative changes of transepithelial electrical resistance (TEER) values in particle exposed Calu-3 cells. Calu-3 cells exposed to solvent alone were defined as 100%.

Cytotoxicity was tested up to 20 µg/insert by determination of dehydrogenase activity (MTS assay), and no decrease in viability of Calu-3 and A549 cells was seen (data not shown). All particles at the chosen concentration of 2 µg/cm^2^ did not influence TEER values of Calu-3 cells at any time (Figure 5).

### 3.4. Particle Uptake by Respiratory Cells in Monoculture (Calu-3 and A549 Cells)

**Figure 6:**
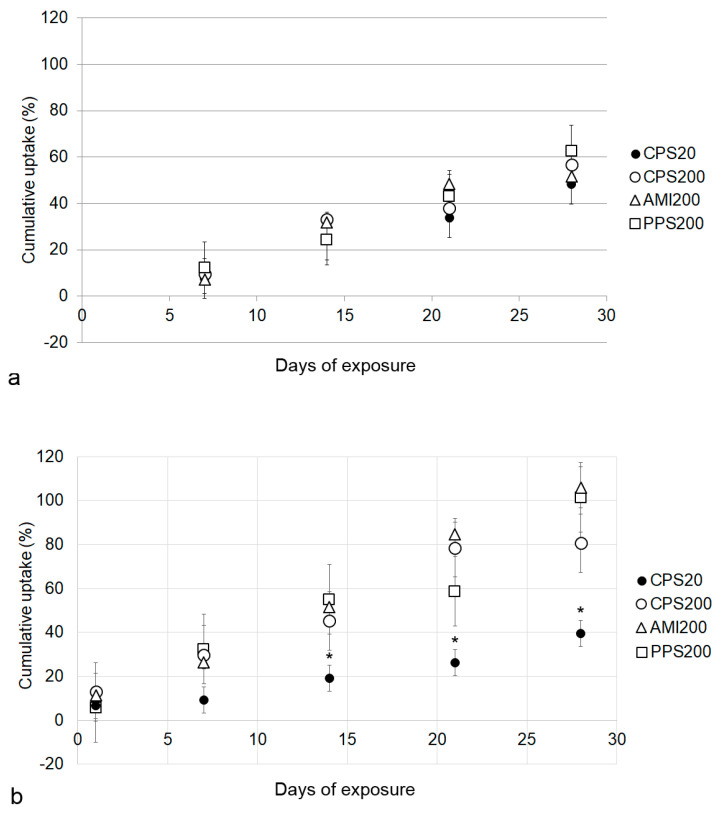
Uptake of polystyrene particles over 28 d by Calu-3 (**a**) and A549 (**b**) cells. The fluorescence of the administered dose is set as 100%. Significant differences are indicated by asterisks.

Both cell lines were compared in order to find out whether the mucus layer formed by Calu-3 cells under ALI conditions had an influence on the particle uptake. The signal in Calu-3 cells increased over the entire exposure time from d7 to d28 (Figure 6a). When cells were exposed for 24 h, however, uptake values were about twice the amount at d7, namely 15.1 ± 2.5% for CPS20, 23.3 ± 1.8% for CPS200, 18.8 ± 5.1% for PPS200, and 26.7 ± 23.4% for AMI200, suggesting some initial adherence to mucus. For the repeated exposures, starting at d7 at each time point, the fluorescence signal increased between 12.1 and 16.6% from one to the next measurement time point (uptakes at each time point divided by the number of doses are listed in Table S1 of the Supplementary Materials).

Particle uptake by A549 cells increased from d1 to d28 by 4.6–21.2% at each time point when suspended in DMEM and by 3.7–14.7% per time point when applied in SLF (Figure 6b). There were no differences noted in the biological reaction between suspension in medium or in SLF. Fluorescence was also measured in the basal compartment to find out if particles crossed the epithelial layer. Maximum fractions of particle fluorescence detected in the basolateral compartment were 0.04% (PPS200), 0.36% (CPS20), 0.13% (CPS200), and 0.06% (AMI200) in the A549 monocultures.

Cell numbers determined at d1–d7, when no dead cells were visible that could interfere with the cell counting (Figure 4), were determined as ~4 × 10^5^ for Calu-3 and ~6 × 10^5^ for A549 cells. Based on the fluorescence in the cumulative uptake, the calculated total amount of ingested particles was ~1 µg/400,000 Calu-3 cells and ~2 µg/600,000 A549 cells.

### 3.5. Particle Effects on Dextran Uptake

Dextran uptake could be visualized in Calu-3 and in A549 cells (Figure S2, Supplementary Materials), but quantification by flow cytometry was only possible for A549 cells. Basal rates of 3 kDa dextran-FITC positive cells were decreased from 4.5 ± 0.3% of total cells at 1d to values between 1.6 and 1.8% at d7–d21 and 0.9 ± 0.7% at d28 (data not shown). The fraction of 10 kDa dextran-FITC-positive cells was 1.7 ± 0.9% of total cells at 1d and between 0.6 and 1.1% at later time points. The corresponding data for 70 kDa dextran were 1.9 ± 0.3% of total cells at 1d and variation of 0.2–0.4% over time.

**Figure 7:**
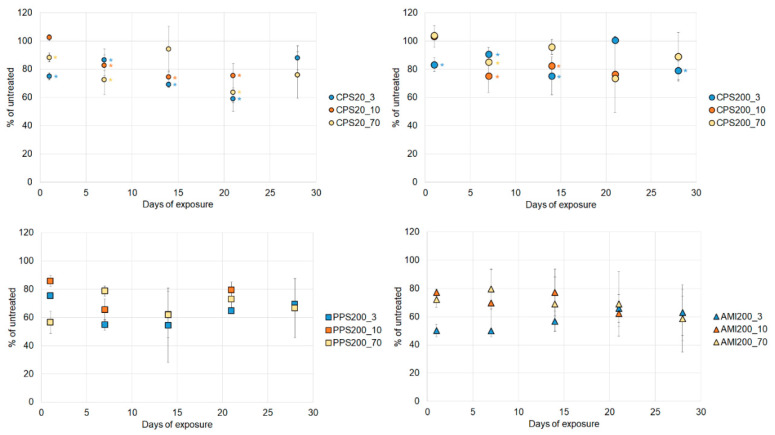
Uptake of 3 kDa, 10 kDa and 70 kDa dextrans by A549 cells. Decrease induced by PPS200 and AMI200 particles were all significant. For exposures to CPS20 and CPS200 particles, significant changes are indicated by asterisks in the respective color.

Particle effects on the uptake showed large variations, which may be attributed to the generally low fraction of cells ingesting the dextrans. Nevertheless, uptake of all dextrans significantly decreased by exposure to PPS200 and CPS200 particles from d1 on (Figure 7). Significant decreases after exposure to CPS20 and CPS200 particles were seen at several, but not all, time points.

### 3.6. Particle Effects on Cytokine Secretion

**Figure 8:**
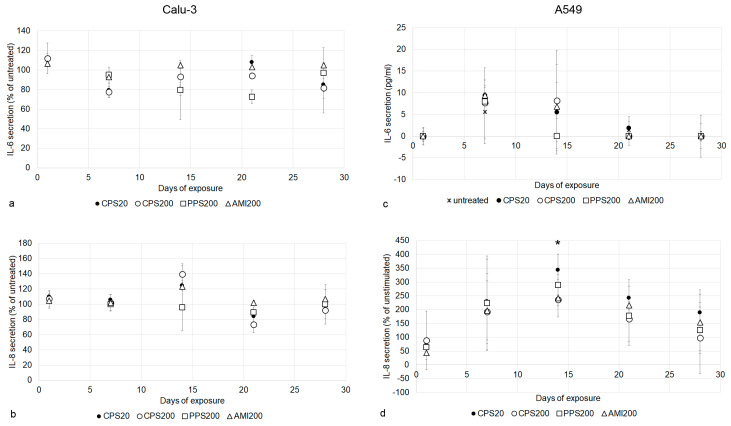
Cytokine levels of Calu-3 (**a**,**b**) and A549 (**c**,**d**) cells upon exposure to particles. Due to values below zero in the controls, IL-6 values for A549 cells (**c**) are indicated in pg/mL, while for the other exposures (**a**,**b**,**d**), relative changes compared to the not particle exposed cells are shown. Significant changes to controls are indicated by asterisks.

All particles did not affect the secretion of IL-6 and IL-8 in Calu-3 cells at any time point (Figure 8a,b). IL-6 secretion by A549 cells was also not influenced in a significant way (Figure 8c). IL-8 levels were increased upon particle exposure between d7 and d21, and the difference to not particle exposed cultures was significant for the CPS20 particles at d14 (Figure 8d).

### 3.7. Particle Effects in A549/THP-1 Co-Cultures

**Figure 9:**
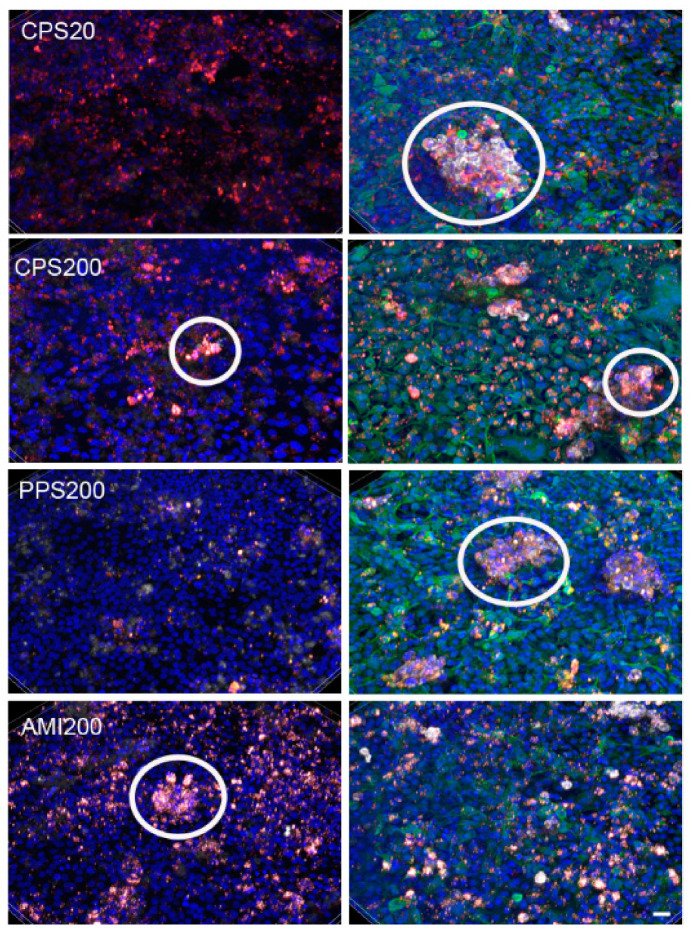
3D projection of z-scan showing particle uptake (red) at d1 and d7 in A549/THP-1 co-cultures. In the left column (d1), only THP-1 cells are stained by CD45 antibody (white). In the right column (d7), in addition to THP-1 macrophages, A549 cells are identified by anti-CK18 immunoreactivity (green). Examples for clusters of CD45-positive THP-1 cells are indicated by circles. Scale bar: 20 µm.

A co-culture with a stable ratio of ~10 A549:1 THP-1 cells was obtained by seeding A549 and THP-1 macrophages in a ratio of 1:1. The 3D projections of the particle-exposed cultures in Figure 9 show that the macrophages were located on top of the A549 cells and often arranged in clusters. These clusters contained higher intensities of nanoparticles than the A549 cells. Quantification of the cell-specific uptake in the co-cultures was not possible by flow cytometry or image analysis due to the difficulties in differentiation between cell types.

At 28 days of A549/THP-1 co-culture, prominent cell death was observed, and the co-cultures did not react to LPS-exposure with an increase in IL-6 and IL-8 secretion, indicating that only data until d21 are reliable. We, therefore, only used data only up to 21 days.

**Figure 10:**
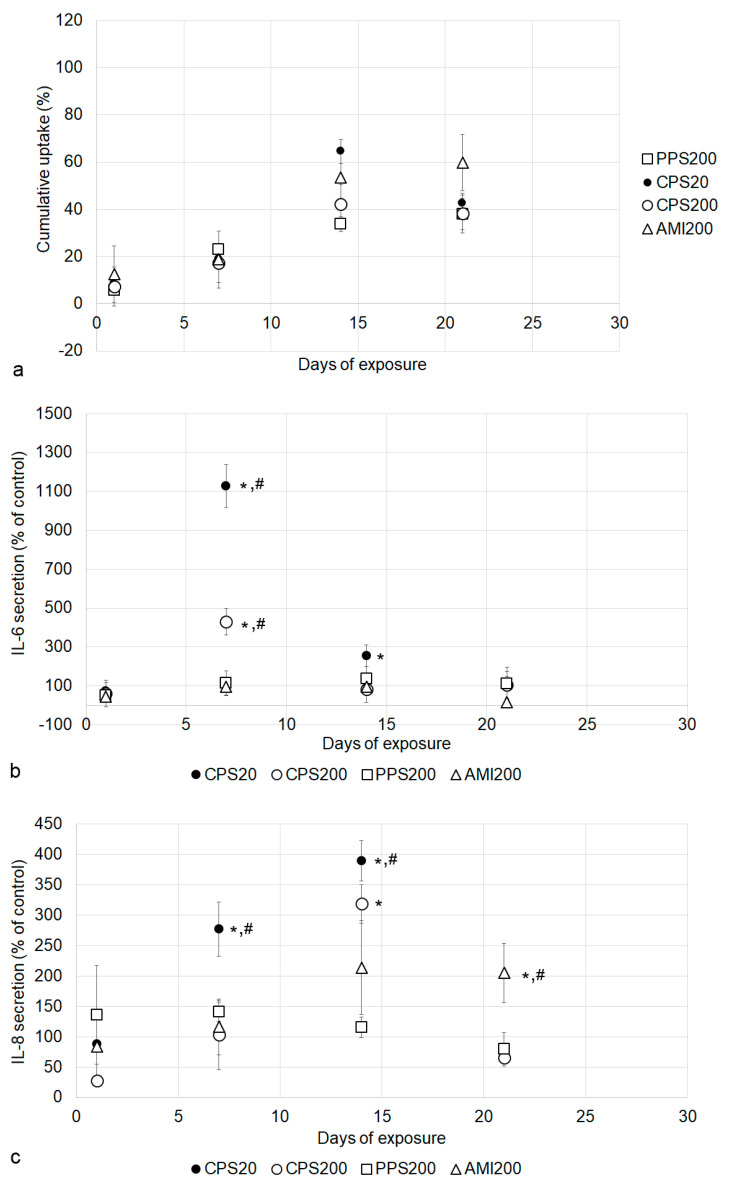
Particle action in A549/THP-1 co-cultures. Cumulative uptake of particles (**a**) and influence on IL-6 (**b**) and IL-8 secretion (**c**) are shown. Significant changes to controls are indicated by asterisks and to other particles by hashtag.

In contrast to the increased fluorescence of the A549 monocultures (Figure 6b), there was no linear particle uptake over time, but the highest fluorescence values were observed at d14 (Figure 10a). Table S1 (Supplementary Materials) shows that the maximum increase in fluorescence, for instance, 21.5% for CPS20 at d14, decreased to 10.6% at d21. Images of the particle-exposed co-cultures suggested that higher concentrations of particles were located in the THP-1 macrophages (Figure S3 of Supplementary Materials). Particles did not permeate the cell layer of the co-cultures, and a maximum amount of 0.12% (PPS200), 0.09% (CPS20), 0.07% (CPS200), and 0.01% (AMI200) of the particle fluorescence was detected in the basolateral compartment of the A549/THP-1 co-cultures. In contrast to the monocultures, where uptake of CPS20 was significantly lower, no significant differences between the particle uptakes were obvious in the co-cultures.

Secretion of IL-6 and IL-8 by the co-cultures differed from the A549 monoculture in the way that CPS20 particles significantly increased secretion of both cytokines at d7 and d14 (Figure 10b,c), while this was only observed for IL-8 at d14 in the monoculture (Figure 8d). Values in the co-cultures induced by exposure to CPS20 particles were significantly higher than the respective interleukin levels induced by the other particles. Increases induced by CPS200 were significantly higher than values of the controls for IL-6 at d7 and for IL-8 at d14. At d21, the AMI200 particles induced significantly higher concentrations of IL-8 than the other particles.

## 4. Discussion

Accumulation of fluorescence as an indication for particle uptake did not reveal inter-particle differences in the exposures of Calu-3 cells. By contrast, A549 ingested CPS200, PPS200, and AMI200 particles significantly more than CPS20 particles. Since the effect was only observed for the CPS20 but not for the larger CPS200 particles, size differences may play a role. The lower uptake of the smaller particles is consistent with previous studies that also reported lower uptake of 20 nm than of 40 nm and 100 nm carboxyl polystyrene particles [26]. Similar findings were also reported for silver nanoparticles [27], where the 20 nm particles were ingested to a lower extent than larger (e.g., 50 nm, or 100 nm) or smaller (e.g., 5 nm) particles. Two facts were proposed to explain the low uptake of 20 nm particles. The 20 nm particles could better diffuse in and out of the cells than larger particles, and the larger particles could employ more uptake routes (e.g., macropinocytosis, clathrin-mediated, caveolae-mediated, and clathrin- and caveolin-independent uptake), while the 20 nm particles are transported only by clathrin-mediated uptake.

The preferential uptake of AMI200 particles is consistent with the generally higher uptake of positively charged particles and was also seen in the conventional culture of A549 cells [28]. By contrast, CPS20 and CPS200 particles were ingested to a higher extent in co-cultures of A549 and THP-1 macrophages, which most likely is due to the preference of macrophages for carboxyl-functionalized particles. Previous data using conventional culture and exposure with the same types of particles showed that nanoparticle uptake by macrophages, in general, was higher than by A549 cells [28]. Further, in the absence of FBS, like in this study, CPS20 particles, followed by CPS200, PPS20 and AMI200 particles, showed the highest rate of macrophage uptake. The preference of carboxyl-functionalized over amino-functionalized particles by macrophages appears to be independent of the particle material because it was reported for 100 nm polystyrene particles and for 25 nm silica particles [29,30,31].

Cellular particle content increased over time for all particles in Calu-3 and A549 monocultures. These data are based on the fact that particle fluorescence was stable over the entire exposure time in the cell culture medium. It may be hypothesized that fluorescence could be decreased due to instability of the polystyrene particles in the acidic environment of the endosomal/lysosomal compartment, where the pH can be as low as 4–4.5 [32]. According to the producers, fluorescent polystyrene particles are stable over a broad range of pH (4–11, e.g., https://www.merckmillipore.com/INTERSHOP/static/WFS/Merck-BE-Site/-/Merck/en_US/PDF/BM-BioMonitoring/IVD/safety-data-sheets/Fluorescent-calibrated-latex-particles-standard-grades.pdf), suggesting that the acid pH should not affect the encapsulated fluorochrome. At d28 Calu-3 cells had ingested ~1 µg of particles/insert with about 4 × 10^5^ cells, while 6 × 10^5^ A549 cells on one insert contained ~2 µg of particles. Based on a cell volume of 2707 µm^3^ for Calu-3 cells and 1204 µm^3^ for A549 cells [33,34], particle concentration was 1 µg/1.08 µL of Calu-3 cytoplasm and 2 µg/0.7 µL of A549 cell volume. The lower particle concentration of the Calu-3 cells may be explained by the hindrance of particle uptake by mucus because Calu-3 cells in ALI culture form a thin, non-continuous mucus layer of about 1 µm [35]. The ingested particle amount of 2 pg/A549 cell at d28 was higher than uptakes reported for 44 nm non-functionalized polystyrene particles of 0.15 pg/oviductal epithelial cell and 0.35 pg/fibroblast [36]. In a previous study, we reported uptake rates after 24 h between 13 pg/cell for CPS20 and 22 pg/ cell for AMI200 particles in A549 upon exposure to 20 µg/mL in conventional culture in the absence of cytotoxic effects [28], suggesting that the cells are able to tolerate higher intracellular concentrations of the particles. The fact that cells did not reach such values upon the repeated exposure in this study suggests a feedback inhibition of uptake or/and excretion of particles. Lysosomal exocytosis has been reported for various types of nanoparticles [37]. The extent to which nanoparticles are excreted depends on the cell type and on the intracellular localization. It appears that mainly nanoparticles contained in endosomes, and not yet released into the cytoplasm, can be exocytosed. Quantification of nanoparticles is complicated and based on analysis of individual cells and short incubation times [38]. Our data on dextran uptake suggest a reduction of endocytosis as a possible mechanism to limit particle uptake. The labeling with 70 kDa dextran is selective for macropinocytosis, while 3 kDa and 10 kDa indicate transport via clathrin-mediated, caveolae-mediated, and clathrin- and caveolin-independent uptake [13]. Dextran uptake values showed marked variation, which may be due to the low basal macropinocytosis rate of epithelial cells. Only 1.3 ± 0.8% of HeLa cells contained 4 kDa FITC dextran after 25 min of incubation according to flow cytometry [39], which is comparable to the basal rate of 4.4 ± 0.3% for 3 kDa FITC dextran in this study. The greatest effect on dextran polymer uptake in this study was detected for PPS200 and AMI200 particles. These particles also showed higher extents of cumulative uptake than the carboxyl-functionalized particles. Particle exposure decreased endocytosis (uptake of 3 kDa dextran) to a greater extent than macropinocytosis (uptake of 70 kDa dextran). It may be hypothesized that polystyrene particles, similar to gold nanoparticles, could inhibit macropinocytosis to reduce intracellular particle concentrations and prevent endoplasmic reticulum (ER) stress [40]. Non-functionalized (plain) and cationic polystyrene particles are preferentially internalized by macropinocytosis [41], which may explain, why uptake of 70 kDa dextran is reduced mainly by these particles and not by the carboxyl-functionalized particles.

Basal (0.04–0.1 pg/mL) and LPS-stimulated (2–5 ng/mL) Il-6 secretion of the A549/THP-1 co-cultures was higher than the respective values (0–1.4 pg/mL and 397–451 pg/mL) of the monocultures. The co-cultures also reacted with higher increases in IL-6 levels than the A549 monocultures to exposure with CPS particles. Basal and LPS-stimulated IL-8 secretion was similar, but the reaction to particle exposure was also more pronounced in the co-cultures. The greater sensitivity of A549 co-cultures to the proinflammatory effect of nanoparticles has also been reported in other studies [42,43,44]. In those studies, hematite and amorphous silica nanoparticles were assessed in epithelial cell/macrophage (A549/THP-1 and A549/Mono Mac 6) co-cultures. Diesel exhaust, titanium dioxide nanoparticles, and single-wall carbon nanotubes were tested in A549/monocyte-derived macrophage/monocyte-derived dendritic cell co-culture and Ag-SiO2 and CuO nanoparticles in A549/THP-1/EAhy926 co-culture. By contrast, Grabowski et al. reported higher basal secretion of IL-6 and IL-8 of A549/THP-1 co-cultures but similar sensitivity in the reaction to PPS200, anatase and rutile titanium dioxide particles [45]. Since different particles have been evaluated in these studies, particle-specific action cannot be excluded. In our study, particle exposure in both A549 and A549/THP-1 cultures caused a transient increase of cytokine secretion, in contrast to the relatively stable secretion in response to LPS. Adaptation of the proinflammatory response to nanoparticles with low cytotoxicity, like 10 nm and 50 nm gold nanoparticles, has been reported in vivo [46]. Upon repeated exposure, the initially induced increase in mRNA expression of proinflammatory cytokines was normalized. Similarly, HaCaT keratinocytes, CHO-K1 Chinese hamster ovary cells, MG63 osteoblast-like cells and EAhy926 endothelial cells showed adaptation to nanoparticle exposure [47,48,49,50]. Mechanisms for adaptation may be a decrease of macropinocytosis as observed for gold nanoparticles [40] or by gene regulation [51]. Genes involved in oxidative stress, apoptosis, DNA damage and inflammation were upregulated upon acute but not chronic exposure of fibroblasts to gold nanoparticles. Gold nanoparticles are not biodegradable and biocompatible and thus may behave similarly to polystyrene particles. The absence of increased cytotoxicity upon repeated exposure to the nonbiodegradable polystyrene particles may indicate that poorly biodegradable carriers for drug delivery like silk fibroin nanoparticles and carbon-based particles may also not accumulate and cause adverse effects upon repeated exposure. It should be noted, however, that the observed cellular behavior has been observed in not inflamed or otherwise stressed cells. Wu et al. reported that in small airway epithelial cells stimulated with tumor necrosis factor-alpha (TNF-α), the ability for adaptation was lost [52]. Animal experiments also suggest that nanoparticles exacerbate pre-existing inflammatory processes of the lung [53].

Our data indicate downregulation of particle uptake and transient proinflammatory response upon repeated exposure to non-cytotoxic concentrations of nonbiodegradable particles. The inclusion of macrophages in the A549 model increased the relative uptake of carboxyl-functionalized particles. Further, the co-culture model reacted more sensitive with cytokine release to particle exposure. The transient nature of the increased cytokine secretion suggests that healthy respiratory cells are able to adapt to low levels of repeated particle exposure.

## Figures and Tables

**Table 1:** Ratios of A549:THP-1 cells at seeding and after 2 and 7 days.

Seeding (A549:THP-1)	2 days (A549:THP-1)	7 days (A549:THP-1)
1:0.4	31.5 ± 6.8:1	8.8 ± 0.6:1
1:1	7.5 ± 2.5:1	11.2 ± 1.2:1
1:1.5	23.5 ± 4.9:1	13 ± 5.6:1
1:2	22.5 ± 2.1:1	8.5 ± 0.7:1

**Table 2:** Particle properties according to producer (functional groups, charge density, size) and hydrodynamic sizes, polydispersity index (PI) and zeta potential of the particles when suspended in cell culture medium (Dulbecco’s modified Eagle’s medium (DMEM) w/o fetal bovine serum (FBS)) and simulated lung fluid (SLF) (PBS + 0.02% dipalmitoylphosphatidylcholine (DPPC)).

Particles	Functionalization (Producer)	Charge (Producer, meq/g)	Size (Producer, nm)	Size (DMEM, nm)	PI (DMEM)	Zeta (DMEM, mV)	Size (SLF, nm)	PI (SLF)	Zeta (SLF, mV)
CPS20	Carboxyl	0.519	24	60	0.07	-31	81	0.25	-23
CPS200	Carboxyl	0.072	200	236	0.05	-48	296	0.08	-11
PPS200	None	n.a.	200	209	0.05	-17	206	0.05	-15
AMI200	Amino	0.087	200	201	0.23	12	757	0.19	5.8

## Supplementary Materials

The following are available online at https://www.mdpi.com/2079-4991/11/3/606/s1, Figure S1: Time-dependent changes in the morphology of Calu-3 cells stained with hemalaun; Figure S2: Staining of Calu-3 and A549 cells with FITC-labeled dextrans; Figure S3: 3D projections of Z-scans of A549/THP-1 co-cultures exposed to polystyrene particles showing particle accumulation after 14, 21, and 28 days of culture; Table S1: Average increase of fluorescence at each time point in Calu-3, A549 cells, and A549/THP-1 co-culture.
