# Supplementary material for: Screening for Effects of Inhaled Nanoparticles in Cell Culture Models for Prolonged Exposure
Source: Nanomaterials (Basel). 2021 Feb 28;11(3):606. doi: 10.3390/nano11030606 (PMC7997552; doi:10.3390/nano11030606)
Supplement: Supplementary file 1 [file nanomaterials-11-00606-s001.pdf]

Supplementary Material

Screening for Effects of Inhaled Nanoparticles in Cell Culture Models for Prolonged Exposure

Table 1. Average increase of fluorescence at each time point in Calu-3 and A549 cells. Fluorescence values were divided by the number of doses that the cells had received at that time point. Abbreviation: 200 nm amine-functionalized polystyrene (AMI200), 20 nm and 200 nm carboxyl-functionalized polystyrene (CPS20 and CPS200), 200 nm non-functionalized plain polystyrene (PPS200), SLF, simulated lung fluid.

Cells (Particle Medium)	Particle	Day 1	Day7	Day14	Day21	Day28
Calu-3 (DMEM)	CPS20	n.d.	7.4±1.8	12.1±0.9	11.2±2.1	12.0±0.2
	CPS200	n.d.	9.4±4.13	16.5±1.5	12.7±1.8	14.1±1.2
	PPS200	n.d.	12.2±2.9	12.2±0.7	14.3±2.1	15.6±3.4
	AMI200	n.d.	7.1±1.1	15.8±1.0	16.1±1.4	12.8±0.7
A549 (DMEM)	CPS20	6.7±1.2	4.6±0.7	6.3±0.6	6.5±0.9	7.8±1.1
	CPS200	12.9±2.9	14.9±5.8	15.0±1.8	19.6±2.8	16.1±2.3
	PPS200	5.7±1.5	16.1±5.0	18.3±5.6	14.6±1.8	20.2±5.0
	AMI200	11.4±1.2	13.2±1.5	17.2±1.8	21.2±1.3	21.2±1.8
A549 (SLF)	CPS20	8.7±3.5	5.5±0.9	11.5±1.7	10.7±0.8	14.7±1.6
	CPS200	13.8±5.0	10.7±8.3	9.9±1.3	10.2±2.0	8.1±2.4
	PPS200	4.2±1.4	3.7±0.6	5.8±1.0	5.5±1.6	7.1±2.6
	AMI200	15.1±2.9	13.5±0.6	12.8±0.8	11.6±1.5	14.4±1.8
A549/THP-1 (SLF)	CPS20	8.5±0.3	9.5±1.0	21.5±4.1	10.6±4.6	n.d.
	CPS200	7.5±1.7	8.6±1.0	14.0±6.0	9.6±1.5	n.d.
	PPS200	9.6±2.1	11.6±4.2	11.2±9.7	9.5±7.4	n.d.
	AMI200	12.6±0.6	9.4±0.9	17.8±1.9	14.9±2.1	n.d.

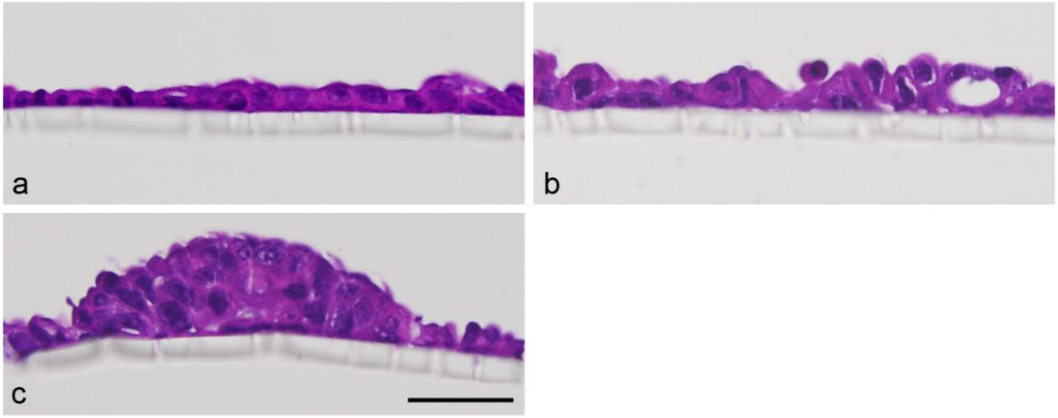

Figure S1. Time dependent changes in the morphology of Calu-3 cells stained with hemalaun. Cells are flatter (a, d7) and more compresses (b, d14). At d21 locations with cell multilayers occur more frequently. Scale bar: 50 µm.

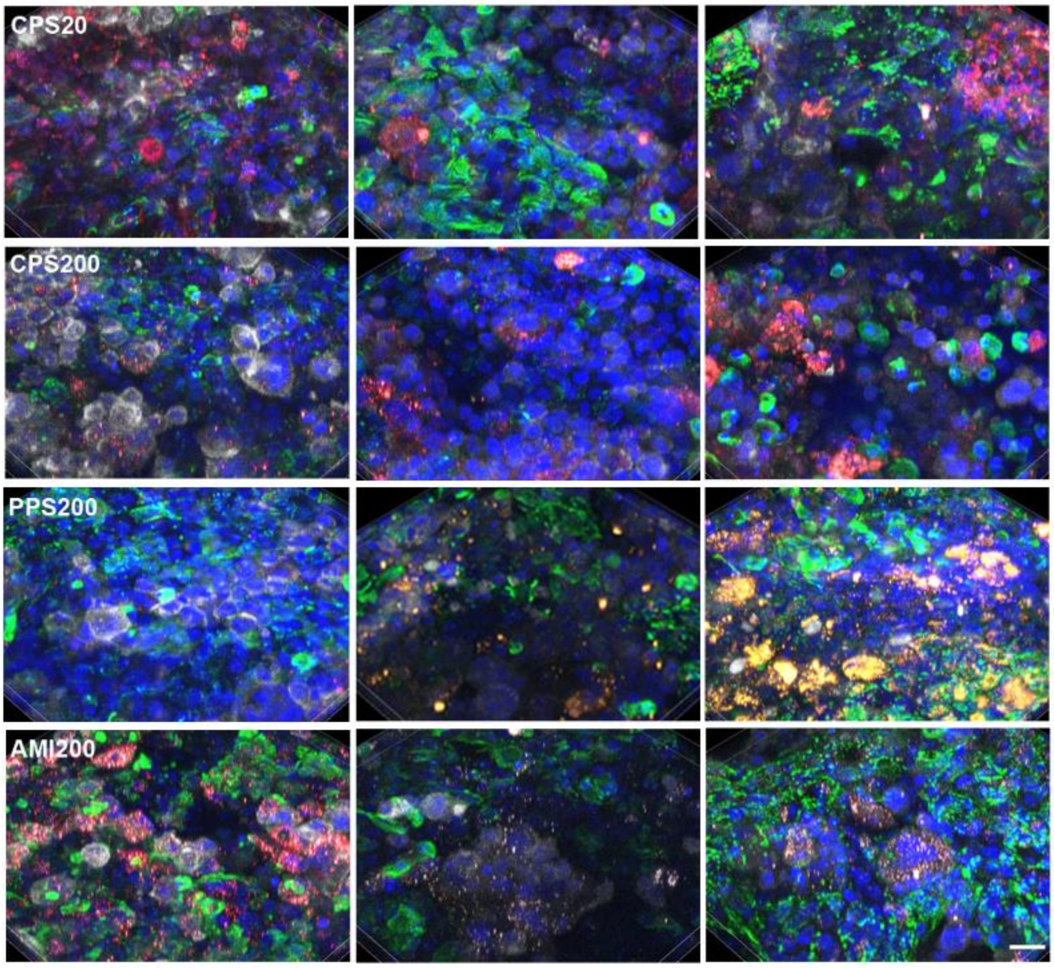

Figure 2. 3D projections of Z-scans of A549/THP-1 co-cultures exposed to polystyrene particles showing particle accumulation after 14, 21, and 28 days of culture. A549 cells are identified with anti-CK18 antibody (green) and THP-1 cells with anti-CD45 antibody (white); polystyrene particles are seen in red. Nuclei are counterstained with Hoechst 33342 (blue). THP-1 cells often occur as clusters. No general difference in the particle uptake pattern over time is obvious, and all particles show higher signals in THP-1 than in A549 cells. Scale bar: 20 μm.

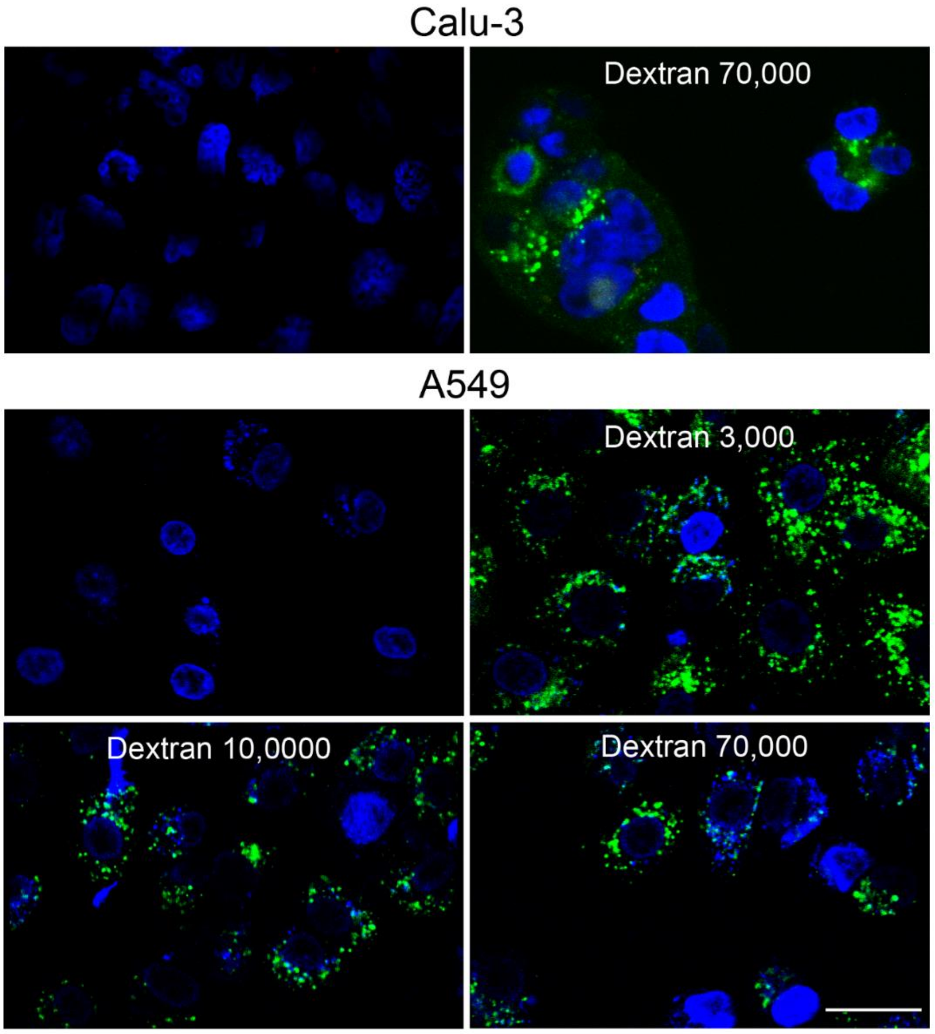

Figure 3. Dextran uptake in not particles-treated cells. Upper panel: Staining of Calu-3 cells in the absence and presence of 70 kDa dextran. Lower panel: A549 without exposure to dextrans and exposure to dextrans of different molecular weight (green). Nuclei are counterstained with Hoechst 33342 (blue). Scale bar: 20 μm.
